# On Morphinism

**Published:** 1880-09

**Authors:** 


### Selections.

### ON MORPHINISM.

We clip the following extract from *The New Orleans Medical and Surgical Journal:*

At a meeting of the Medical Society in Berlin, Dr. E. Levinstein presented some remarks on the above mentioned disease, based upon his late experience.

As to the question whether it be preferable to institute a sudden or gradual breaking of the habit of using morphine, the author has several times before pronounced himself in favor of the abrupt method, and he prefers it still as a rule, inasmuch as it often happens that serious symptoms appear when the gradual breaking of the habit is finally accomplished; in employing the abrupt method these symptoms usually cease in 4–5 days. The abrupt method is, however, very potent, and delicate persons, especially women, often have difficulty in enduring it; medical attendance must be on hand continually for the sake of assistance in case of necessity. In patients suffering from chronic, painful and incurable diseases, the abrupt method is not to be carried through. In such patients where death is evidently near on hand a breaking of the habit is entirely out of the question, but in other cases, where it is impossible to calculate life's duration, it is undoubtedly in the patient's interest to get rid of his habit, as morphine finally ceases to give relief even when used in steadily increased dose. The nervous system is, so to speak, saturated and cannot be acted upon in the same manner as before; the toxical effect of morphine only remains, while soothing, alleviating effect does not appear any longer. But when the constitution, through a breaking of the habit, has been purged of morphine, the former normal condition returns and quite a small quantity of morphine has again a narcotic action. In patients suffering from incurable, painful diseases we ought consequently to institute a breaking of the habit in order to enable them to be benefitted by the use of a remedy, which, when used in the proper way, may give ease and soothe the pain, but when abused, only can increase the sufferings in adding a fresh one.

In such patients Dr. L. has employed a modified method, which mainly depends upon an abrupt breaking of the habit and he has succeeded with it in patients suffering from phthisis, emphysema, heart disease, etc.

The modified method is described in the following way: in order to ascertain the quantity of morphine the patients are consuming daily, they are, previous to the actual treatment, isolated and put under observation, and for 2 or 3 days they get the dose of morphine they have indicated themselves; after that all morphine is stopped suddenly. According to the author's experience it is only exceptionally and only when the patient's nutrition has not been properly attended to, that a serious collapse occurs during the first 24 hours after the last hypodermic injection. But after the lapse of this period the critical symptoms appear, which may develop into a dangerous collapse; as soon as this is threatening to occur (tardy pulse, slow and irregular respiration, violent diarrhœa and vomiting), we have to do something to provent it; if the patient formerly has been using very large doses (45 to 60 grains during 24 hours), 1-30 of this amount will be sufficient to produce if not a very pleasant condition, at any rate a tolerable one and make the worst symptoms disappear; if the daily dose has been 7½ to 15 grains, 1-15 of this amount will prove sufficient, and if not more than 7½ grains a day have been used, 1-10 of the usual dose will do.

It is advisable to make the patient commence the cure at night time; by this arrangement the discomfort is light during the first night; during the following day the symptoms caused by the abstinence, increase and reach a considerable height towards night; but at this point a moderate hypodermic injection is administered to the patient and makes him pass an endurable night; the next night a somewhat diminished injection is given (1-40, 1-20, 1-15 of the usual dose), and so forth the 3d, 4th, 5th day until the dose has been reduced to 1-6 ½ grain, these doses as a matter of course ought not to be imitated unconditionally; in serious cases the physician must be guided by circumstances; in few instances, however, will he find it necessary to allow larger doses.

The author has used this modified method also in delicate persons and only in few cases did he have to give any injection after the first two nights.

Through the modified method we have learned, that already in a short time after having made the patient stop using morphine, a small dose of it is sufficient to prevent the breaking out of violent symptoms and we possess in it a remedy to facilitate considerably the troubles, which are started by the entire and abrupt discontinuance of using morphine; Dr. L. intends, however, yet to use the latter method in robust individuals, whilst the modified method is indicated in women, delicate persons and in patien's, where the consumption of morphine has to be reduced to a minimum.

Taking in consideration the advantages attached to the modified rapid breaking of the habit, nobody will probably adhere to the gradual discontinuance; it is, however, advisable to watch the patient as closely as in using the abrupt method, although the task has been rendered much easier to the patient as well as to the physician and the nurse.

When a person has been cured of morphine and has recuperated his former health in body and mind, he is generally exposed to suffer a relapse, and we must prepare to protect him against this occurrence.

Is it after all, possible to prevent the relapses? Under certain conditions, yes; but besides possessing considerable power of will, it is necessary that the individual should not be too far advanced in age, that his pecuniary position should allow him to make some sacrifice and that he should not have used very large doses of morphine for a great number of years.

When a patient successfully has accomplished his cure and is on the point of being dismissed, he ought to be told, that although cured of morphinism, of morphine poisoning, he is not yet clear of all danger, and that during the first year he will be subject to attacks during which it will be difficult for him to resist the desire for morphine; it will consequently be to his own interest willingly to submit himself to a careful control, in order to be protected against a relapse; if he be unmarried, care should be taken that he does not live alone but together with a friend. Druggists, as a rule, relapse immediately, and there is nothing to be done for them if they cannot sacrifice their position; the same is the case with physicians, if they do not abstain from administering injections of morphine personally to their patients.

This sounds very harsh and seems to violate the dignity of man; is it possible that men, occupying so high an intellectual position, should possess no more power of will and control over themselves? And yet experience shows it to be so. In many cases the physician could administer the remedy (morphine) to his patients in some other shape in place of the hypodermic injection, and where the injection seems absolutely necessary, he will have to employ a remplacant whom he may instruct and superintend; still better would it, however, be to turn the patient over to a confrere. Hypodermic injections of morphine administered by a physician disposed to morphinism rouse in him the desire for morphine, and from thought to action there is but a short distance, when the desire for drug is involved.

The author points out that a certain (fortunately limited) number of morphiophagi can not do without morphine forever; this was the case in certain individuals who had consumed 15 to 30 grains of morphia a day for a number of (10 to 15) years; they did not suffer from any actual disease of body and mind, but they did not feel satisfied being without the use of morphine; with a healthy appearance they had recuperated their appetite and sleep, and yet they did not feel at ease; 5 to 6 months later they lost their appetite, became sleepless, lost flesh and wasted away. No actual disease was to be discovered and in the beginning the author thought that they had relapsed and were using morphine clandestinely, but by close observation he satisfied himself that this was not so. On the other hand it was found that these very rarely occurring cases are cured just by morphine, which had become an absolute necessity to the constitution by being used for years; it would be acting on a wrong principle out of dogmatism to persevere in refusing morphine to this class of patients; before morphine is allowed to them, the symptoms should, however, be carefully investigated. As a rule there is nothing to be feared from total abstinence of morphine during the first 5 to 6 months in old individuals, and during the first year in middle aged persons; if then a decline sets in, we ought to interfere and allow a little morphine; at times a vital indication may demand it; 1-12 to 1-6 gr. given 2 or 3 times a day is, however, sufficient, and it is not even necessary to increase the dose to procure sleep, appetite and comfort.

The author has had under treatment 110 patients suffering from morphinism; 82 of them were men, 28 women. The preponderance of the men is probably caused by their social position.

Amongst the 82 men and 28 women, 32 were physicians, 8 wives of physicians, 1 the son of a physician, 1 a medical student, 2 charitable sisters, 2 male nurses, 1 midwife, 6 druggists, 1 the wife of a druggist—54 consequently belonging to the medical profession and its appendices—army officers are represented by 18 men and 1 woman, of the balance 11 were merchants, 5 wives of merchants, 4 wives of civil officers, 2 single ladies, 3 male and 2 female rentiers, 3 landed proprietors, 4 lawyers, 1 male and 2 female teachers. The youngest was 21 years old, the oldest 65 years.

Twenty men and 6 women had acquired the habit of using subcutaneous injections of morphine through acute diseases, 46 men and 17 women from chronic diseases complicated with pain and discomfort, 1 man had used the remedy as an antaphrodisiacum, 15 men and 5 women had been induced to use morphine partially in order to put themselves in a pleasant mood, partially in order to forget domestic troubles. Out of the 110 patients 12 men took to drinking while using morphine. Of the 82 men 61 relapsed, of the 28 women 10 did so. Of the 32 physicians 28 relapsed.

In looking for the cause of the comparatively large number of physicians addicted to morphine and especially exposed to a relapse, we come to the conclusion that their profession is the cause of it; it is not the desire of enjoyment that induces them to use morphine. The author has treated most respectable men for this disease, men who were enthusiasts in their profession, of very unselfish disposition, and who just disregarded themselves entirely in the fulfillment of this duty towards their patients. Often it happens that a physician, through a painful disease, having been accustomed to the use of injections of morphine, has to resume his practice a little too early; and as he can by no means appear weak and faint before his patients and yet has to stand exertions that demand health in body and mind, he seizes the remedy that already has made him get over many painful hours.

The relapses are in several cases depending on the effect caused by an immoderate use of morphine for a long time.

Months after the patient has been deprived of morphine, when he seems to be entirely restored to health, fear and restlessness appear at a sudden, repeating itself in some days, and these attacks are complicated with a strong desire of morphine; these attacks may occur isolated, but sometimes they occur for days and weeks so violently that the patient succumbs to his desire if he be not possessing an extraordinary firmness, or if the necessary precautions have not been adopted.

Finally, the physicians are often to be blamed; when a patient has been successfully treated for morphinism and later happens to be suffering, or simulates to be suffering, from some painful trouble (tooth-ache, sick head-ache) the attending physician administers sometimes to such a patient an injection of morphine, which in many cases causes a relapse to their former passion (amongst 8 patients which the author had got under treatment a second time, the relapse had originated in this manner in 5.)

Morphinism is of very little importance in criminal law, as it is hardly possible that a crime committed by a morphiophage can ever be excused by lack of responsibility. In civil law morphinism may be of some importance and sooner or later the life insurance companies will find it necessary to take a certain position towards morphinism as well as towards alcoholism. There is no doubt that morphiophagi as well as alcoholists shorten their lives and in case of life insurance inflict damage on the company. Morphinism can, however, not be looked upon in the same light as alcoholism, as nobody turns alcoholist from therapeutical causes, while the morphine consumer usually has a therapeutical object, intending to soothe pain, sleeplessness or fright; even when abused it often has to be considered as a medicant; the life insurance companies can hardly help taking this in consideration; only those individuals, who, without suffering from any disturbance of body or mind, make an abuse of morphine, may be looked upon in the same way as drunkards.

The insurance companies, as is well known, have by-laws, stating that the proprietor of a policy cannot claim the insurance money, when the insured person (whether sane or insane) caused his death by suicide; it is consequently entirely indifferent whether the party were imputable or not. In the latter years the author has had occasion to get acquainted with the death of 4 physicians who were morphiophagi; 3 of them had used morphine in order to alleviate morbid conditions; they gradually became accustomed to it and had to increase the dose to obtain effect, until they finally reached a dose which proved fatal; they went to bed apparently in their ordinary condition and presented the next morning symptoms of acute morphine poisoning, which in a few hours caused death in spite of all treatment; they had killed themselves, certainly unintentionally, but this according to the above is of no consequence. As to the fourth confrere something similar happened to him; he tried in his home to submit himself to treatment; but this experiment failed as usually is the case, and he got hold of 1-3 of his usual dose for 24 hours; a dose which 8 days before hardly would have had any narcotic effect, but which now caused death. The modified method has shown us that when the patient has been deprived of morphine for 24 hours, 1-15 of the usual dose is sufficient to check and even to overcome the violent symptoms following abstinence. Through abstinence the system is in a very short time purged of morphine, and its narcotic effect can display itself as before when given in a small dose; under such circumstances death may occur from a dose which before had been comparatively innocuous. Whether these 4 cases ought simply to be dealt with according to the insurance companies' rule on suicide is a question of law. At any rate the companies will find themselves compelled to take a decision in regard to morphinism when issuing new policies. In controversial questions the heirs of the insured parties will have to go to the courts, who will have to procure the opinion of physicians conversant with the subject and decide the individual case accordingly.—(*Berliner Klin. Wochenschr.*, 1880.—*Ugeskr. f. Læger.*

Dr. E. Levinstein has on several former occasions given accounts of his experience with morphinism. He points out that the disease is of modern origin, dating from the introduction of the hypodermatic syringe into general practice. The originators of the disease are those physicians, who, treating more or less painful and tedious diseases, have left it to the patients to administer on themselves the injection.

Morphinism presents similar symptoms as alcoholism (delirium, fright, trembling, hallucinations and diminished resistance in case of an accidentally occurring disease); they differ by the fatty degenerations missing in morphinism, that mania does not occur and finally that the victims of morphinism nearly exclusively belong to the higher classes in society.

For those persons who quite often inject morphine on themselves, this remedy finally becomes indispensable, in the same way as liquor to the habitual drunkard; it makes them get over their low spirits, forget their domestic unhappiness, their troubles in business and so on; they obtain, however, merely temporary relief, and have to have recourse to their poisonous remedy to be alleviated in their melancholy condition.

Characteris ic for morphinism it is that sugar is often found in the urine of such patients; it disappears, however, usually in a few days, when the patient ceases to use morphine. Sometimes, also, albumen is found in the urine, but it takes months to make that disappear.

As to the prognosis Dr. L. remarks that out of quite a large number of cases, he has hardly seen 25 per cent. cured; most of them relapsed; 2 cases turned out fatal from marasmus, 2 patients committed suicide; in these 4 cases the patients did not want to submit themselves to a regular treatment. Five patients became addicted to drinking, amongst them a physician's wife, who had learned from a pharmacology that alcohol was the antidote of morphine, and a remedy to break one of the habit of using morphine. She followed the advice and took to the opposite extreme.

The treatment consists chiefly in withdrawing the morphine from the patient's use. It is preferable abruptly to stop the supply of morphine, as the system is better able to endure an energetic interference than a slow and tedious action as we often see it to be the case in surgery and obstetrics.

In pronounced cases it is out of question to break the patients of their habit of using morphine, except they be entirely treated like prisoners. They have to be isolated and continually to be under the custody of refined and incorruptible persons. It is difficult to find such assistants, as some of them clandestinely will supply the patient with morphine out of covetousness, and others are unable to resist their entreaties and the sight of their hard suffering. Doors and windows must be scrupulously secured from all communication with the outside world; clothing and utensils belonging to the patient must be carefully searched, it being invariably the case that every patient carries a considerable amount of morphine along with him and one or more syringes, on entering the institution in order to be cured of morphine, whether it be spontaneously or against his will. The physician can in no way rely on such a patient; promises, the most solemn protestations, the patient's word of honor, which is given without the slightest hesitation, all is without any importance, morphine disparaging the character of man, as all passion does. The most refined and otherwise most considerate and intellectual men, do not shun any means or any ruse to deceive the physician in order to get back the morphine they brought along or procure some fresh morphine. If the physician be energetic, if he observes continually his patient, if he has honest assistants, and if he watches them closely also, the worst part of the cure will be passed in 8 days.

Usually, 12 hours after morphine has been withdrawn a collapse occurs; it is for this reason better to let the patient stay in bed, and during the first 8 days to be quite liberal in giving stimulating and fiery wines. Even women need during this period to be stimulated by alcoholic drinks in considerable quantity. The collapse may reach such a degree that it becomes dangerous to life; the danger may be averted by a very small injection of morphine. If the patient does not wail during the first 48 hours of the abstinential period, if he be able to eat his meals in the course of the first days, and if he continues to look well, he has, in spite of all denying it, clandestinely taken morphine. The persevering narrowness of the pupil (if this symptom did exist previously) and the absence of diarrhœa will soon corroborate this surmise.

The patient's despair and restlessness is during the first 3 days so violent, that the physician must be thoroughly determined to do his duty, to be able to resist the patient's crying and despair. The patient must be well protected against attempts of committing suicide, as his miserable mental condition may easily induce him to do so. Bathing in warm water is used with advantage to relieve the neuralgies that appear during the abstinence, also to induce sleep at night; if the collapse be not too serious, cold water may be poured over the patient after the bathing. The diarrhœa which, according to L.'s experience, always occurs immediately after the withdrawal of morphine, requires treatment only when it is exhausting him too much; large injections of warm water into the rectum have proven beneficial. Continued vomiting may necessitate feeding the patient by clysmata.

Although alcoholic drinks are indicated, their quantity should be moderated as soon as the patient is able regularly to take his meals, as there exists a close relationship between morphinism and alcoholism. Fresh air, substantial food and preparations of iron will soon assist in recuperating the lost strength. It is furthermore of importance, already in the third week of the treatment, to let the patient have some occupation of his body and especially of his mind, to rouse his self-esteem and make him conscious that his faculty is not destroyed.

Experience shows that the internal use of morphine and the hypodermic injection, as long as administered by the physician himself, does not lead to morphinism, but that the disease has been developed only when the physicians commenced to leave the administration of the injection to the patients themselves, to mid-wives and to nurses. It is often stated in defense of this bad habit, that the physician may be prevented from giving the injection himself; if so, he must content himself with giving morphine internally; given per anum, or on an empty stomach, morphine is just as much pain-subduing and narcotic as by a subcutaneous injection, although the action may be a little more slow. By giving it internally, the euphory which is just the item that makes morphine fascinating, is missing, and the patient can very well do without that. Our only remedy to check morphinism from being further propagated, is that the physicians in future keep the morphine-syringe under their own control exclusively.—*Berliner Klin. Wochenschr.*, 1875.—*Hosp. Tid.*

